# Cutting edge for technical textiles (fluorescent, antibacterial and UV-protective) by incorporation of thienoisoquinoline-quinazoline derivatives

**DOI:** 10.1186/s13065-025-01504-3

**Published:** 2025-06-19

**Authors:** Walid Sharmoukh, Islam S. Marae, Etify A. Bakhite, Hanan B. Ahmed, Hossam E. Emam

**Affiliations:** 1 Department of Inorganic Chemistry, National Research Centre, 33 EL Buhouth St., Dokki, 12622 Giza Egypt; 2 Department of Chemistry, Faculty of Science, Assiut University, Assiut, 71516 Egypt; 3 Chemistry Department, Faculty of Science, Helwan University, Ain-Helwan, Cairo, 11795 Egypt; 4 Department of Pretreatment and Finishing of Cellulosic Based Textiles, Textile Research and Technology Institute, National Research Centre, Scopus Affiliation ID 60014618, 33 EL Buhouth St., Dokki, 12622 Giza Egypt

**Keywords:** Quinazoline derivatives, Cotton, Fluorescence, UV-protective, Durability

## Abstract

Nowadays design of multi-functionalized textiles as technical textiles is highly demanded to be applied in different environments and under hard weather condition. Herein, tri-functionalized (fluorescent, antimicrobial and UV-protection) cotton textiles were designed by immobilization of heterocyclic compounds based on quinazoline derivatives (**QDs**). Firstly, four quinazoline derivatives (**QD-1 = H, QD-2 = O–CH**_**3**_**, QD-3 = Cl, QD-4 = H** [**without COOH**]) were synthesized for the first time starting from 2-(chloromethyl) quinazoline-4(3H)-one. The chemical structure of all obtained **QDs** were investigated by NMR (^1^H & ^13^C) and infrared spectroscopy, in addition to the measurement of melting points, yields and emission spectra. Secondly, the synthesized **QDs** were immobilized within cotton textile, while cotton was per-activated by interaction with cationic reagent. The modified textiles (QDs@Q-cotton) gained dark yellow color. The QDs@Q-cotton emitted greenish radiation and showed intense emission fluorescence at 485–521 nm. Good–very good (UPF = 23.6–32.8) and good (21.3–25.8) UV protection was respectively shown for QDs@Q-cotton before and after 10 washings. The protection from UV radiation for QDs@Q-cotton is attributed to the reflection of UV radiation by effect of **QDs** micro particles which deposited within the cotton matrix. The modified textiles exhibited antimicrobial action against *S. aureus* and *E. coli* bacteria, while the mortality was 79.9–89.1% and 69.7–76.2% before and after 10 washings, respectively. The antibacterial activity of the QDs@Q-cotton fabrics is attributed to the **QDs** skeleton. The highest antibacterial action for QD-3@Q-cotton is related to the chlorine derivative. The multi-functionalized textile with good durability could be successfully employed in the military/soldiers clothes.

**Supplementary Information:**

The online version contains supplementary material available at https://doi.org/10.1186/s13065-025-01504-3.

## Introduction

Technical textiles are defined as category from textiles that manufactured for technical or functional purposes rather than decorative or aesthetic purposes. Functional textiles are specialized for designing to incorporate number of smart characters which permit them to be applied in specific purposes and consequently deeply contribute to expand their application scope. The most used technical or functional textiles are fluorescent/photo-luminescent textiles [1, 2], UV-protective textiles [3–8], self-cleaning [1, 9], antimicrobial or medical textiles, [7, 10–12], insect repellent [13, 14], water/oil repellent textiles [1, 9, 15], flame retardant [16–18], electrical conductive textiles [19–21], electromagnetic blocking [22–24] and highly sorbent textiles [25, 26].

Fluorescent and photo-luminescent textiles are one interesting type from the technical textiles which are changing their color due to exposure to the ultraviolet radiation/light or can emit radiation under dark environment (glow in the dark) [27–29]. Due to such unique optical properties, designing of fluorescent textiles are highly demanded nowadays, while can be employed in the army/military textiles, soldier’s clothes, police man clothes, sporting goods, paper currencies and toys [27]. Based on the former studies, different materials are used to design photo-luminescent textiles including fluorescent dye or pigment [27, 28, 30–36], conducting polymers [37], metal organic frameworks [1, 2], composite containing strontium [38, 39] and nanomaterials such as carbon dots and nanogold [40–43].

On the other hand, antimicrobial and ultraviolet (UV) protective textiles which known as protective textiles, are classified as one important applicable class from the technical textiles. Antimicrobial (medical) textiles are mainly applicable in the hospital (such as surgical gowns, patient clothes, gloves, masks, sheets, blankets and curtains) in order to control the infections’ spreading [44, 45]. Additionally, bandage, wound dressing and gauze are a non-woven medical textile commonly used within the body of human [46]. The global market of medical textiles is covered by 14.4 billion dollars in 2020 and predicted to increase to 19.7 billion dollars during 2026 [47]. The widely used microbicide reagents in textile industry can be organic and inorganic reagents. Chitosan, quaternary ammonium salt, triclosan, polyhexamethylene biguanide, N-halamines and carbon nanostructures are the most utilized organic microbicide reagents [8, 12, 48–50]. The inorganic microbicide reagents presented in metal oxides metal nanoparticles (Ag, Au, Pd) and metal organic frameworks [7, 43, 51, 52]. While, the UV protective textiles are applied for outdoor clothes and in hot weather environment to be protect from the harmful effect of solar radiation. Many materials are considered as UV radiation blocking including dyes, metallic nanoparticles, metal oxides and metal organic frameworks [2, 5–7, 10, 43, 51–54].

Various types of heterocyclic compounds were reported to be successfully applicable for preparation of multifunctional textiles [55–59]. Quinazoline compounds are bicyclic hetero compounds composed from the pyrimidine moiety and benzene ring [60]. Compounds containing quinazolines are widely displayed as bioactive compounds such like as anti-bacterial [61, 62], antioxidant [63], analgesic [64], antiviral [65], anti-inflammatory [64, 66, 67], antimalarial [68, 69], anti-diabetic [70] and anti-cancer [71–73]. Most of aromatic polycyclic compounds are signified with fluorescent material, corresponding to the delocalization of electrons in aromatic system which can be easily excited [74]. Moreover, heterocyclic compounds based on nitrogen are shown to be importantly applicable for design of chemo-probe [75]. Therefore, quinazoline derivatives (QDs) as heterocyclic compounds were synthesized herein and subsequently, incorporated with cotton for formulation of multi-functionalized (fluorescent, antimicrobial and UV-protection) textiles.

## Experimental section

### Materials and chemicals

Anisaldehyde (≥ 97.5% purity (GC), liquid), benzaldehyde (≥ 98%, FG, FCC), acetylacetone (≥ 99%), chlorobenzaldehyde (97%), sodium acetate trihydrate (ACS reagent), (≥ 99%), sodium acetate anhydrous (> 99%, FG), piperidine (Reagent Plus^®^, 99%), sodium metal (99.95%), sodium hydroxide (ACS reagent, ≥ 97.0%, pellets), sodium silicate (reagent grade), dimethyl sulfoxide (DMSO) (≥ 99.9%), chloroform (CHCl_3_) (≥ 99.8%), methanol (≥ 99.8%) and hydrogen peroxide (35%) were all supplied from Sigma-Aldrich with analytical grade and used as received without purification.Cyanothioacetamide and chloromethylquinazolien were synthesised and purified according to the reported methods**.** Bleached woven cotton fabrics (180 gm/m^2^) purchased from Misr Company for Spinning and Weaving, El-Mehalla El-Kobra—Egypt.

### Synthesis of thienoisoquinoline-quinazoline derivatives

#### Synthesis of 2-(1-amino-5,8-dimethyl-6-phenyl-6,7,8,9-tetrahydrothieno[2,3-c]isoquinoline-2-yl)quinazoline-4(3H)-one; compound [QD-(1–3)]

In a round-bottle flask, 7-acetyl-6-hydroxy-1,6-dimethyl-8-phenyl-3-thioxo-2,3,5,6,7,8-hexahydroisoquinoline-4-carbonitrile derivatives [compound **(a1-3)**] (10 mmol), 2-(chloromethyl) quinazoline-4(3H)-one [compound **(b)**] (10 mmol) and sodium acetate (20 mmol) were all dispersed in 150 mL of ethanol and leaved to reflux for 2 h. By reaction time, the yellow color of the mixture was gradually disappeared and returned back. After 1 h the yellow precipitates of compounds (**QD1-3**) were precipitated and isolated by filtration. The obtained yellow precipitates were washed one time with water and two times with hot ethanol to remove the un-reactant stuck. The obtained solids were air dried prior to analysis and reuse. The yield of obtained compounds (**QD-1**), (**QD-2**) and (**QD-3**) were 4.3 g (84%) and 4.9 g (90%) and 4.9 g (90%), respectively. The melting points of compounds (**QD-1**), (**QD-2**) and (**QD-3**) were 289–291 °C, 298–300 °C and 294–296 °C, respectively.

#### Synthesis of 2-(1-amino-5,8-dimethyl-6-phenyl-6,7-dihydrothieno[2,3-c]isoquinoline-2-yl)quinazoline-4(3H)-one; compound [QD-4]

A mixture of 2-(chloromethyl) quinazoline-4(3H)-one [compound (**b**)] (10 mmol) and 1,6-dimethyl-8-phenyl-3-thioxo-2,3,7,8-tetrahydroisoquinoline-4-carbonitrile [compound (**d**)] (10 mmol) with sodium acetate (20 mmol) was suspended in 150 mL of ethanol and then refluxed for 2 h. The yellow color of the reaction was gradually disappeared then returned with the time. After 1 h, the yellow precipitate of compound (**QD-4**) was precipitated. The yellow precipitate was filtered out and washed with water once followed by washing by hot ethanol remove the un-reactant stuck. The obtained precipitate was dried on air prior to analysis and reuse. The compound **(QD-4)** was obtained with yield of 4.3 g (84%) and its melting point was 237 °C.

### Modification of cotton by thienoisoquinoline-quinazoline (QD) derivatives

#### Cationization process of cotton

In order to activate the fabrics through increase the reactive functional groups, the cotton fabrics were cationized by interaction with the quaternary ammonium salt as previously reported [42, 43, 54]. Firstly, fabrics were alkalized by padded through sodium hydroxide solution (2 N). In the second step, alkalized cotton was padded in 3-Chloro-2-hydroxypropyltrimethyl ammonium chloride (50%, wt/vol) and squeezed to get a 100% wet pick-up. The treated fabrics (Q-cotton) were thermos-fixed at 150 °C for 5 min followed drying 120 °C for 10 min. The obtained cationized cotton (Q-cotton) were neutralized with 1% acetic acid and then washed two times with tap water. The fabrics were then dried at 80 °C prior to using.

#### Loading onto cotton

The synthesized compounds (**QD 1–4**) were loaded onto the cationized cotton (Q-cotton) fabrics by using dipping process. A 0.5 g of the synthesized compounds (**QD 1–4**) were dispersed in 50 mL of chloroform (CHCl_3_). Specimens of fabrics (0.5 g) were separately submerged in the four different synthesized solutions for 15 min under stirring. The specimens of fabrics were taken out followed by thermofixation and drying at 140 C for 3 min prior to characterization. The modified fabrics were named as QD-1@Q-cotton, QD-2@Q-cotton, QD-3@Q-cotton and QD-4@Q-cotton.

### Characterization and analysis

Thin-layer chromatography (TLC) analysis was conducted using aluminum sheets measuring of 2.5 × 5 cm, coated with silica gel (60F-254) at a thickness of 0.25 mm. Visualization was achieved through iodine and UV light exposure. Silica gel (60–120 and 100–200 mesh) was utilized for column chromatography. A Gallan-Kamp device was employed to determine the melting points of compounds and the results reported without correction.

The prepared compounds were analyzed using various spectroscopic techniques. A Shimadzu 470 IR-spectrophotometer (KBr) was employed to obtain infrared (IR) spectra. Jeol-Ex-300 NMR spectrometer (JEOL, Tokyo, Japan) and chemical shifts were expressed as part per million; (δ values, ppm) against TMS as an internal reference, National Research Center, Cairo, Egypt. ^1^H and ^13^C chemical shifts were referred to the solvent signal (DMSO-*d*_*6*_) at 2.50 and 39.52 ppm, respectively. Data are presented as follows: chemical shift, integration, multiplicity (s = singlet, d = doublet, dd = doublet of doublet, td = triplet of doublet) and coupling constants in Hertz (Hz). NMR was conducted using a 500 MHz spectrometer for ^1^H NMR and a 126 MHz spectrometer for ^13^C NMR. Measurements were taken at 25 °C using CDCl_3_ or DMSO-d_6_ as solvents. For ^1^H NMR, internal standards were set at 7.26 ppm for CDCl_3_ and 3.33 ppm for DMSO. In ^13^C NMR, internal standards were 77.00 ppm for chloroform and 39.50 ppm for DMSO-d_6_. Tetramethyl silane (TMS) served as the reference standard at 0.00 ppm. Peak patterns were classified as singlet (s), doublet (d), doublet of doublet (dd), triplet (t), quartet (q), or multiples (m). Coupling constants (J values) were reported in hertz (Hz). An Elementar Analyses system GmbH VARIOEL V2.3 1998 CHNS Mode was used for elemental analyses.

The surface properties and morphology of the modified cotton (QD-1@Q-cotton QD-2@Q-cotton, QD-3@Q-cotton and QD-4@Q-cotton) were examined under the high resolution scanning electron microscope (Quanta FEG 250 with the field emission gun, from FEI Company—Netherlands), as the surface coated with a gold layer before testing. The elemental characterization of fabrics was measured using the analyzer of energy dispersive X-ray (EDAX AME-TEK analyzer). The spectral of infrared were detected for the modified fabric (QD-1@Q-cotton QD-2@Q-cotton, QD-3@Q-cotton and QD-4@Q-cotton) via using the Jasco FT/IR 6100 spectrometer. The spectral data was measured in the transmission mode and collected in the wavenumber range of 4000–400 cm^–1^ using resolution of 4 cm^–1^ and 64 scanning times with 2 mm/sec scanning rate.

The color measurements parameters (L*, a*, b*, yellowness index [YI] and color strength [K/S]) were all measured for the modified fabrics (QD-1@Q-cotton, QD-2@Q-cotton, QD-3@Q-cotton and QD-4@Q-cotton) using the UltraScan Pro spectrophotometer (Hunter Lab, from USA). The parameter of color coordinates of L*, a* and b* are corresponding to the lightness [black (0)/white(100)], the red (+)/green ratio (−) and the yellow (+)/blue ratio (−), respectively [10, 11]. The measurements of each sample were performed three times at different areas and the mean values were addressed. The tensile strength of the modified fabrics (QD-1@Q-cotton, QD-2@Q-cotton, QD-3@Q-cotton and QD-4@Q-cotton) were tested by using Asano machinery, MFG—Japan, according to the standard method of “ASTM method D2256-66T”.

### Optical properties

The absorbance spectra for the solution of compounds (**QD-1, QD-2, QD-3 and QD-4**) were measured by using FLAME-S-UV–VIS spectrometer. Fluorescence emission for the compounds (**QD-1, QD-2, QD-3 and QD-4**) and the modified fabrics (QD-1@Q-cotton QD-2@Q-cotton, QD-3@Q-cotton and QD-4@Q-cotton) were estimated at the excited wavelength of 360 nm by Jasco FP-6500 spectrofluorometer, while 150 W Xenon lamp was used with the concave holographic grating.

The digital photos of the modified fabrics (QD-1@Q-cotton QD-2@Q-cotton, QD-3@Q-cotton and QD-4@Q-cotton) were obtained in closed box containing UV lamp with power of 4 W and the input current was 220 V AC. The photos were picked at excitation wavelength of 265 nm and 325 nm by cell phone camera of OPPO A31 model with 12 MP.

### UV protection

The ultraviolet radiation (UVR) protection was performed for the modified fabrics (QD-1@Q-cotton QD-2@Q-cotton, QD-3@Q-cotton and QD-4@Q-cotton) through measurement the UVR transmission (T %) by using the JASCO V-750 spectrophotometer—from Japan in the UV transmission range of 280–400 nm. The UV protection in UV-A range (UVA-315–400 nm), UV protection in UV-B range (UVB, 280–315 nm) and total UV protection factor (UPF) were all estimated according to the AATCC test 183–2010.44 method. The estimation was carried out twice at different areas and the mean values were concerned.

### Antibacterial activity

The antibacterial activity for the all modified fabrics was tested against two different pathogens using the standard quantitative method of shaking flask test according to the literature [76, 77], while, the bacterial reduction was estimated. The antibacterial performance was tested for two strains represented in Gram-negative bacterium *of Escherichia coli* (*E. coli,* ATCC 11775) and Gram-positive bacterium of *Staphylococcus aureus* (*S. aureus,* ATCC 12600). The method is performed as follows: the pathogenic strains were inoculated at 37 °C on the nutrient agar. The bacteria were grown until reach 10^8^ colony-forming unit (CFU)/Ml. Consequently, a 10 µl from bacterial solution was added to 5 Ml of the liquid NB media which supplemented with 100 µg from cotton sample. The samples were placed on a 200 rpm rotary shaker and incubated at 37 °C for 12 h. The growth in bacteria was measured via detecting the optical density (OD) at maximum wavelength of 550 nm using the spectrophotometer, while the control broth without samples was used as blank. The bacterial reduction in percentage was estimated through the difference in optical densities between the blank and the sample.

### Durability

The wash-fastness of the modified fabrics (QD-1@Q-cotton, QD-2@Q-cotton, QD-3@Q-cotton and QD-4@Q-cotton) against the repetitive washing was examined up to 10 washing cycles. The washing process was carried out according to the standard home laundry test method of AATCC [78]. The modified fabrics were soaked in the washing solution [containing sodium carbonate (2 g/L) and commercial detergent (1 g/L)] at 55 ± 3 °C using material to liquor ration of 1:100 under stirring. After 15 min, fabrics were removed, squeezed and then rinsed with the tap water for 5 min and then dried at 80 ± 3 °C to get the first washing cycle. The washing process was 10 times repeated to obtain 10 washings.

## Results and discussion

### Synthesis

**Fig. 1:**
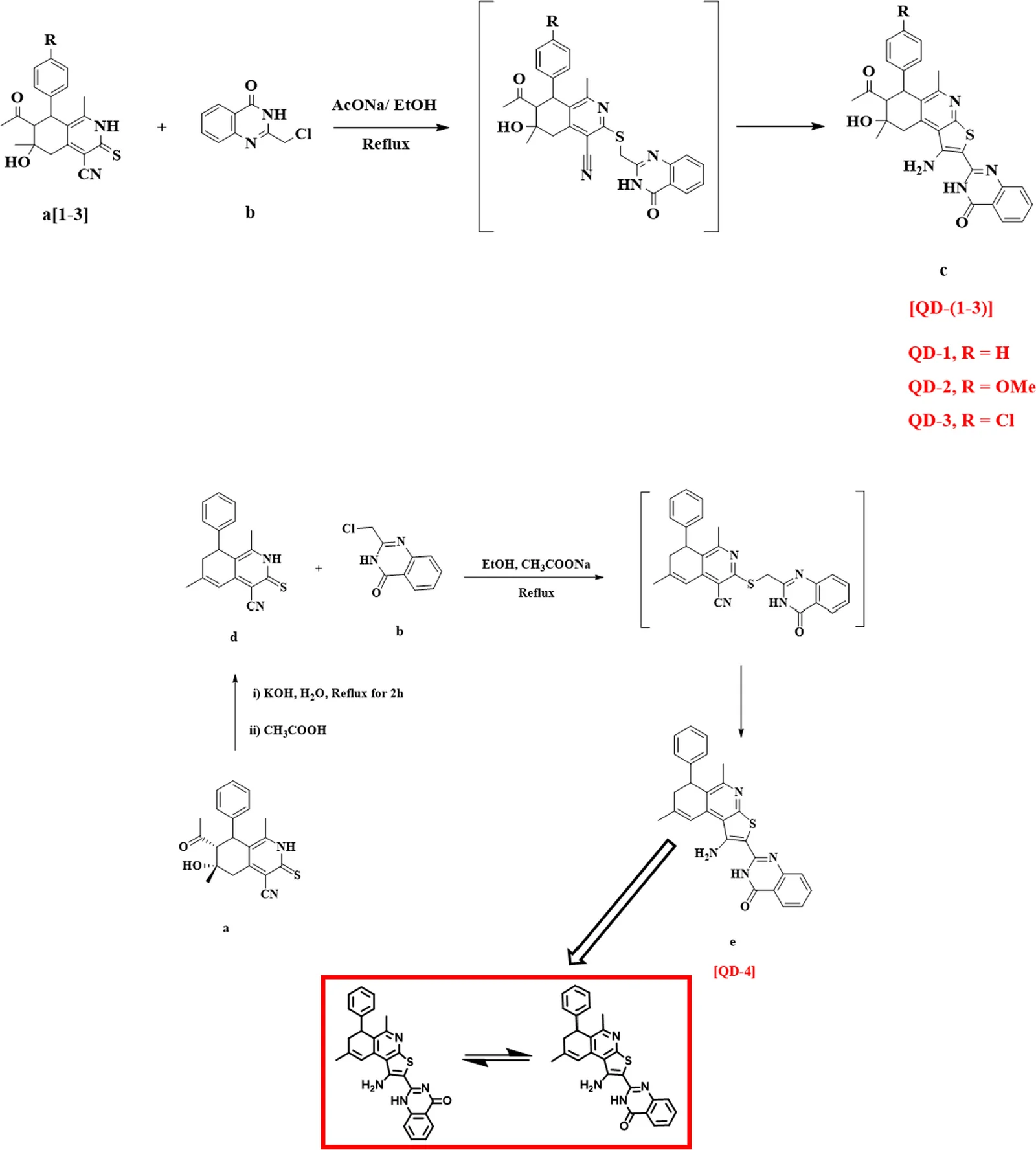
The preparation scheme of quinazolines derivatives (QDs)

Four quinazolines derivatives (QDs) were synthesized starting from 7-acetyl-6-hydroxy-1,6-dimethyl-8-phenyl-3-thioxo-2,3,5,6,7,8-hexahydroisoquinoline-4-carbonitrile derivatives [compound (**a1-3**)] with different derivatives and 1,6-dimethyl-8-phenyl-3-thioxo-2,3,7,8-tetrahydroisoquinoline-4-carbonitrile [compound (**d**)]. Compound (a1-3) and compound (d) was individually interacted with 2-(chloromethyl)quinazoline-4(3H)-one (compound b) in presence of sodium acetate under refluxing conditions as schematic in Fig. 1.

Compounds **a1–3** and **d** reacted individually with chloromethylquinazoline through SN2 alkylation reaction forming thioether derivatives while sodium acetate was used as soft base under continuous refluxing condition in an ethanol. Subsequently, an intramolecular Ziegler cyclization was successfully carried out producing the corresponding thieno[2,3-b]isoquinoline derivatives (**QD-1–4**), as depicted in Fig. 1. Notably, compound QD-4 was found to exist in two distinct conformations, as evidenced by its analysis using ^1^H NMR and ^13^C NMR spectroscopy.

### Spectral analyses

**Fig. 2:**
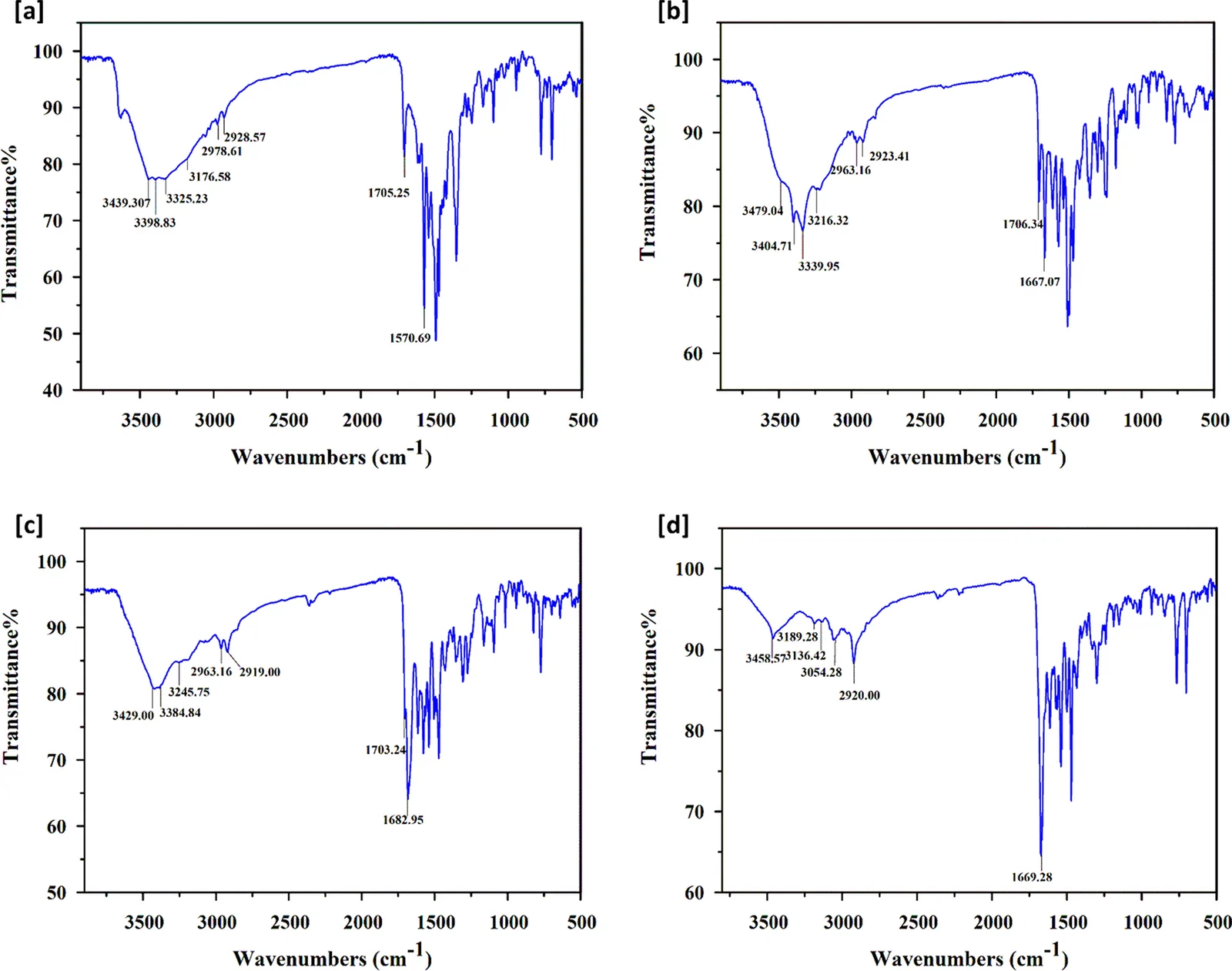
FTIR spectra of the synthesized QDs; **(a)** QD-1, **(b)** QD-2, **(c)** QD-3 and **(d)** QD-4

The chemical structure of the synthesized compounds of **QD-1, QD-2, QD-3** and **QD-4** were determined by the spectral analyses of ^1^HNMR (supplementary data, Figure S1) and infrared (Fig. 2). The infrared spectra of **QD-1** compound exhibited characteristic transmission peaks at 3427 cm^−1^ (OH), 3272 cm^−1^ (NH), 3056 cm^−1^ (aromatic C–H), 2944 cm^−1^ (aliphatic C–H), 1804 & 1902 cm^−1^ (phenyl group) and 2224 cm^−1^ (C≡N). While, for **QD-2**, distinctive bands were recorded at 3479 cm^−1^ (OH); 3404 (NH), 3339–3216 cm^−1^ (NH_2_); 3050 cm^−1^ (aromatic C–H); 2963, 2923 cm^−1^ (aliphatic C–H); 1706 cm^−1^ (C=O, in acetyl group) and 1667 cm^−1^ (C=O, in amide group). In case of **QD-3,** bands at 3429 cm^−1^ (OH); 3384 (NH), 3245–3216 cm^−1^ (NH_2_); 2963, 2919 cm^−1^ (aliphatic C–H); 1703 cm^−1^ (C=O in acetyl group) and 1682 cm^−1^ (C=O in amide group) were clearly obtained. The characteristic bands of **QD-4** shown at 3458 cm^−1^ (NH), 3189–3139 cm^−1^ (NH_2_); 3054 cm^−1^ (aromatic C–H), 2919 cm^−1^ (aliphatic C–H,) and 1669 cm^−1^ (C=O of amide group).

The ^1^H NMR for **QD-1** compound showed signals at *δ* of 11.76 (s, 1H, NH), 8.08–8.09 (d, 1H, *J* = 7 Hz, Ar–H), 7.86 (bs, 1H, –NH_2_), 7.78–7.81 (t, 1H, Ar–H), 7.63–7.65 (d, 1H, *J* = 7 Hz, Ar–H), 7.39–7.42 (t, 1H, Ar–H), 7.23–7.26 (t, 2H, Ar–H), 7.18–7.19 (t, 1H, Ar–H), 6.98–7.00 (d, 2H, Ar–H), 4.72 (s, 1H, OH), 4.62–4.64 (d, 1H, *J* = 10 Hz, CH-Ph), 3.63–3.66 (d, 1H, *J* = 17 Hz, CH_2_ of aliphatic ring), 3.41–3.45 (d, 1H, *J* = 17 Hz, CH/CH_2_ of aliphatic ring), 2.88–2.90 (d, 1H, *J* = 10 Hz, CH-Acetyl), 2.14 (s, 3H, CH_3_ of acetyl group), 2.01 (s, 3H, CH_3_ of pyridine ring) and 1.32 (s, 3H, CH_3_ of aliphatic ring). The ^13^C NMR for **QD-1** declared **22** environments for carbon at *δ* of 210.42, 158.81, 157.16, 148.44, 145.34, 142.80, 135.25, 130.02, 129.16, 128.57, 126.48, 126.48, 67.83, 67.53, 66.75, 40.42, 40.26, 40.08, 31.85, 28.53, 25.13 and 24.70 ppm.

The summarized ^1^H NMR signals for **QD-2** compound were *δ* = 11.76 (s, 1H, NH), 8.07–8.09 (d, 1H, *J* = 8 Hz, Ar–H), 7.85 (bs, 1H, –NH_2_), 7.79–7.81 (t, 1H, Ar–H), 7.63–7.65 (d, 1H, *J* = 7.5 Hz, Ar–H), 7.39–7.42 (t, 1H, Ar–H), 7.30–7.32 (d, 2H, *J* = 8.5, Ar–H), 7.20–7.36 (d, 2H, *J* = 8.5, Ar–H), 4.79 (s, 1H, OH), 4.65–4.67 (d, 1H, *J* = 10.5 Hz, CH-Ph), 3.62–3.65 (d, 1H, *J* = 17.5 Hz, CH_2_ of aliphatic ring), 3.41–3.45 (d, 1H, *J* = 17.5 Hz, CH/CH_2_ of aliphatic ring), 2.84–2.86 (d, 1H, *J* = 10.5 Hz, CH-Acetyl), 2.18 (s, 3H, CH_3_ of acetyl group), 2.02 (s, 3H, CH_3_ of pyridine ring) and 1.32 (s, 3H, CH_3_ of aliphatic ring). While 15 different carbon environment were recorded in ^13^C NMR at *δ* = 210.24, 144.36, 131.39, 130.47, 129.11, 126.48, 123.68, 99.98, 67.74, 40.52, 40.36, 40.19, 31.60, 28.74 and 25.14 ppm.

In case of **QD-3** compound, the recorded ^1^H NMR signals were at *δ* 11.76 (s, 1H, NH), 8.07–8.09 (d, 1H, *J* = 8 Hz, Ar–H), 7.80 (bs, 1H, -NH_2_), 7.77–7.80 (t, 1H, Ar–H), 7.63–7.65 (d, 1H, *J* = 7.5 Hz, Ar–H), 7.39–7.42 (t, 1H, Ar–H), 6.70–6.91 (d, 2H, *J* = 8.5, Ar–H), 6.80–6.81 (d, 2H, *J* = 8.5, Ar–H), 4.71 (s, 1H, OH), 4.56–4.58 (d, 1H, *J* = 10.5 Hz, CH-Ph), 3.70 (s, 3H, O-CH_3_), 3.61–3.64 (d, 1H, *J* = 17.5 Hz, CH_2_ of aliphatic ring), 3.39–3.42 (d, 1H, *J* = 17.5 Hz, CH CH_2_ of aliphatic ring), 2.84–2.86 (d, 1H, *J* = 10.5 Hz, CH-Acetyl), 2.15 (s, 3H, CH_3_ of acetyl group), 2.02 (s, 3H, CH_3_ of pyridine ring) and 1.31 (s, 3H, CH_3_ of aliphatic ring). The ^13^C NMR spectra showed 22 different carbons at *δ* = 210.59, 158.90, 158.04, 157.06, 142.60, 137.04, 130.33, 129.59, 126.49, 125.72, 123.64, 114.52, 114.23, 100.03, 67.84, 66.93, 55.52, 40.25, 40.08, 31.80, 28.54 and 25.19 ppm.

The obtained **QD-4** compound showed several ^1^H NMR signals at *δ* = 12.45 (s, 1H, NH), 11.75 (s, 1H, NH), 8.04 (bs, 1H, Ar–H), 7.74 (bs, 1H, -NH_2_), 7.77–7.80 (t, 1H, Ar–H), 7.58 (bs, 1H, Ar–H), 7.37 (bs, 1H, Ar–H), 7.25 (bs, 1H, Ar–H), 7.18 (bs, 1H, Ar–H), 7.14 (m, 8H, Ar–H), 6.981 (bs, H, Ar–H), 6.46 (s, 1H, *SP*^2^-CH), 4.28–4.34 (d, 2H, CH-Ph), 2.91 (bs, 2H, CH_2_), 2.27 (ds, 6H, CH_3_) and 1.83 (ds, 6H, CH_3_ of pyridine ring). While, 22 signals were appeared in the ^13^C NMR at *δ* = 159.76, 158.75, 158.38, 154.25, 149.60, 149.08, 145.65, 144.43, 142.87, 134.98, 129.14, 128.94, 127.75, 127.52, 127.24, 127.03, 126.32, 99.65, 31.10, 24.90, 24.62 and 22.38 ppm.

### Optical properties of QDs

**Fig. 3:**
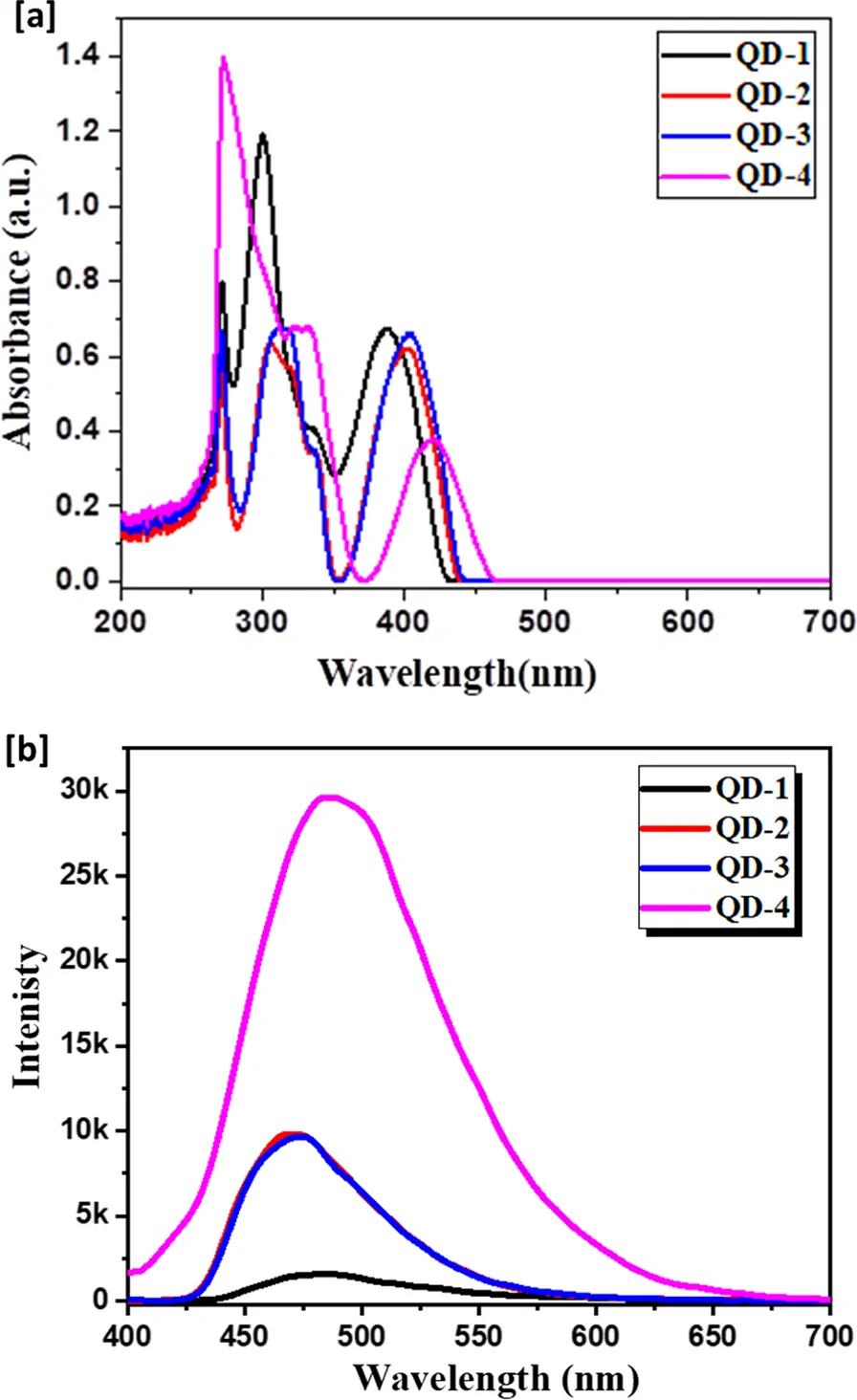
Optical properties for the synthesized QDs; **(a)** UV–vis spectra and **(b)** Emission spectra (at 360 nm)

Solutions of the synthesized **QDs (QD-1, QD-2, QD-3** and **QD-4)** were visually seen with yellow color. According to the previous studies, increasing of the conjugation leads to shift the absorbance to much longer wavelength in the visible region (< 380 nm) and consequently the compound showed color [79]. Three absorption bands were clearly observed for the four synthesized **QDs**. The measured absorbance spectra (Fig. 3a) for the four compounds showed three absorption peaks at 264–271 nm, 303–334 nm and 388–422 nm. The reported peaks are characterized for the n-σ*, π–π* (e.g. C=C) and n-π* (e.g. C=O, C=N), respectively [79–82]. Compared to **QD-1, QD-2** and **QD-3** exhibited absorbance at longer wavelength because of the presence of the electron withdrawing groups of chloride and methoxy substituent. Due to the removing of acetyl and hydroxyl groups from the starting compound, **QD-4** compound showed blue shift and the the obtained peaks were detected at slightly longer wavelengths.

In general, the absorbance of the synthesized **QDs** compounds are minly corresponding to their fluorescence character which may be related to the inter-molecular bondings of the compounds with heteroatoms of S and N atoms [82, 83]. The fluorescence emission spectra were obtained for the the synthesized **QDs** compounds after the excitation at 330 nm as preesnted in Fig. 3b. The excited **QDs** compounds exhibited a significant fluorescence peak at 440–485 nm, which corrospodning to the greenish area as reproted in the previous works [84, 85]. The fluorescent charater of the **QDs** compounds was attributed to the elecron transistion of π–π* / n-π* in overlapping with the substitutents of function groups. The flourecence intensity followed the order of **QD-4** > > > **QD-3** ≥ **QD-2** > > **QD-1**. The flourescence inensity of **QDs** could be explianed by the substituted groups which did not share in the congugation system and resulted in increment the fluorescence intentsity. Comparing with **QD-1**, the highr intensity of flourescence for **QD-2** and **QD-3** is related to the presence of substituted groups of electron withdrawing (Cl & O-Me) [86, 87], which helped in increasing the propability of intersystem crossing and subsequently increase emission intensity. While, **QD-4** compound showed the highest fluorescence intensity might be related to the absence of electron donating groups (O=C–CH_3_ and OH).

### Application onto textiles

**Fig. 4:**
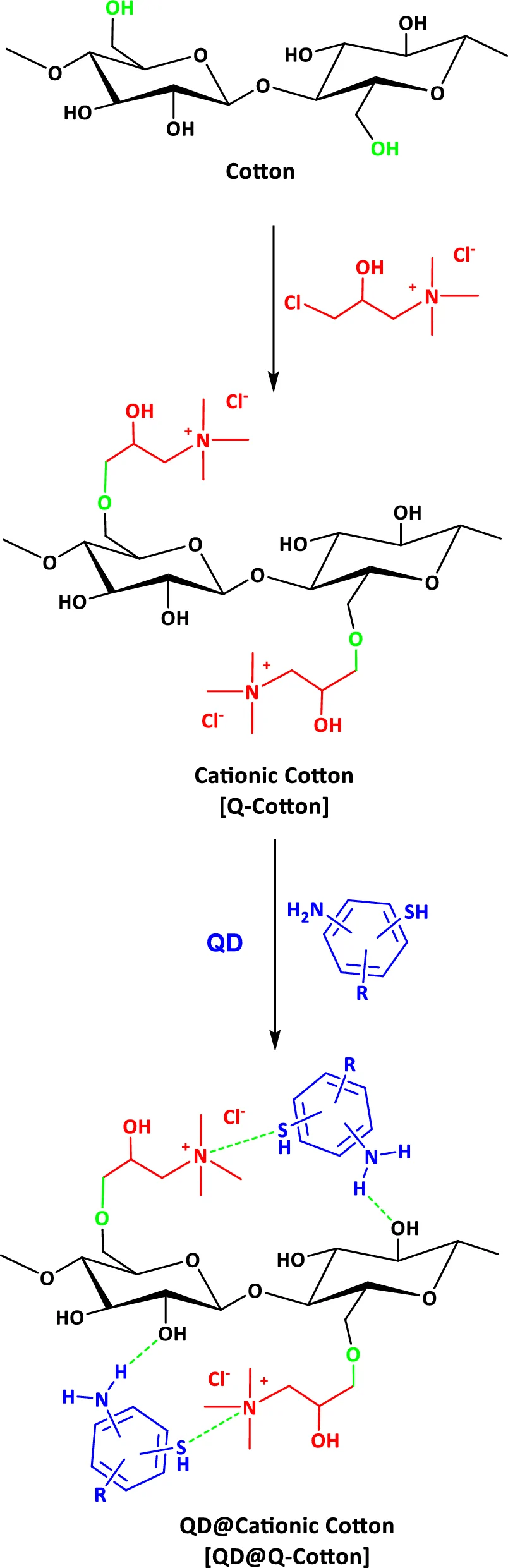
Modification of cationic cotton with the synthesized QDs

The four obtained quinazolines derivatives compounds **(QDs 1–4)** were loaded onto the cotton fabrics. Firstly, cotton samples were activated through functionalization with the quaternary amine producing cationized cotton (Q-cotton) as shown in Fig. 4. Sodium hydroxide activated the cotton fabrics by dissociation of the primary alcoholic groups of cellulose as well as the swelling of cellulose fibrils which in turn permit the interaction with cellulose [2, 12, 42, 43]. The quaternary amine of 3-Chloro-2-hydroxypropyltrimethyl ammonium was interacted with the dissociated alcoholic group at C-6 of cellulose through a substitution reaction forming Q-cotton [2, 12, 42, 43]. Secondly, the Q-cotton is chemically interacted with **QDs** via weak interactions presented in hydrogen bonding and van der Waals force [88]. Hydrogen bonding may form between the hydroxyl and amine groups in Q-cellulose with the different functional groups of **QD** such as OH, NH, NH_2_, O, N, S [15, 25, 89]. In addition to the chemical interaction, QD could be physically immobilized within the intermolecular spaces and pore of the fabrics [7, 25].

**Fig. 5:**
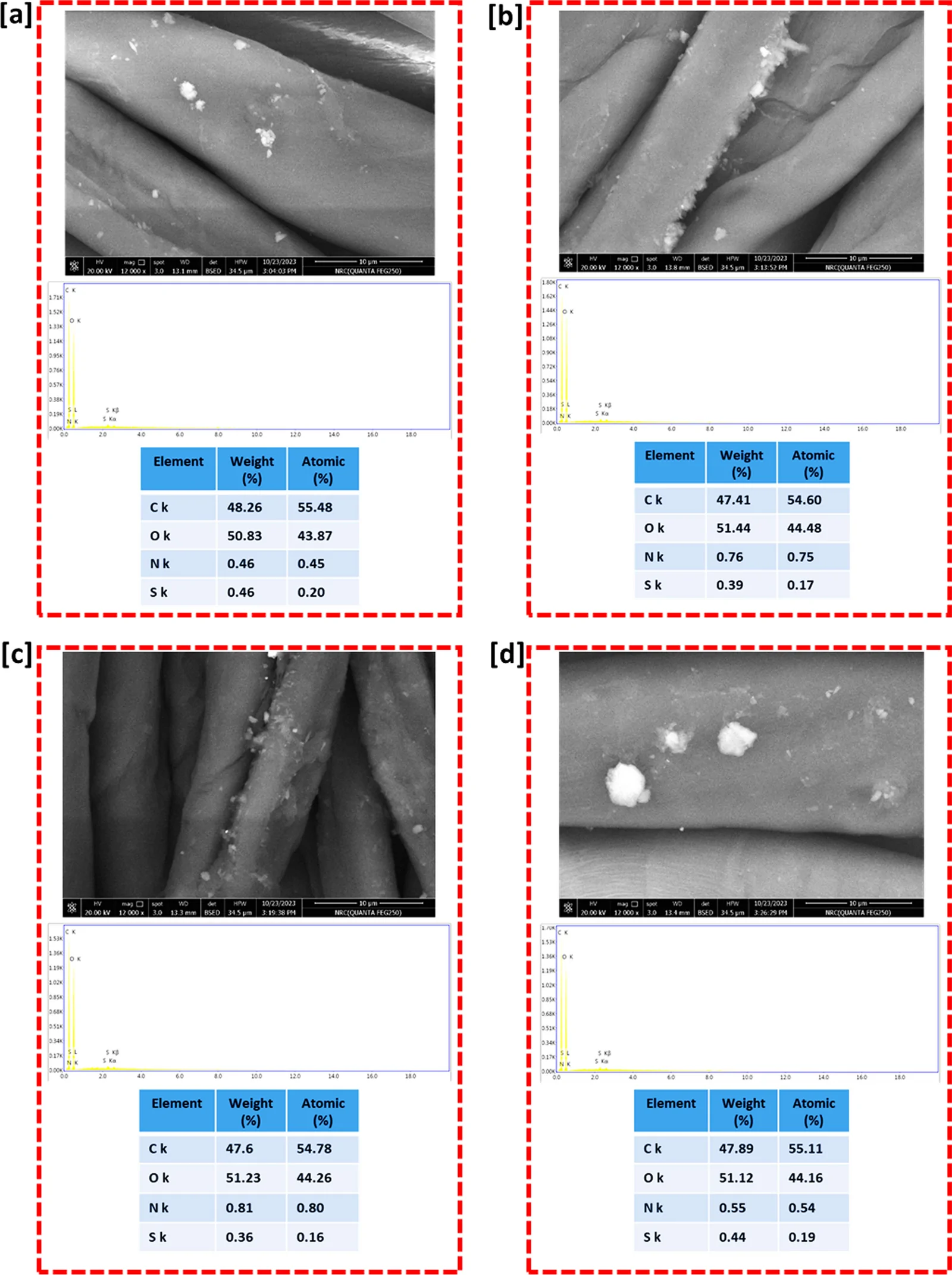
Scanning electron micrographs for QDs@Q-cotton fabrics; **(a)** QD-1@Q-cotton, **(b)** QD-2@Q-cotton, **(c)** QD-3@Q-cotton and **(d)** QD-4@Q-cotton

The morphological characters and the elemental analysis for the surface of modified fabrics were examined as seen in Fig. 5. For all modified fabric samples, the signals for elements of C, O, N and S were obviously recorded which proved the modification of fabrics with **QDs**, while, the signals of N and S are related to **QDs**. From the elemental analysis, the weight percentage of N of S elements were in the same range (0.36–0.81%) which suggests that the loaded amounts of **QDs** onto fabrics are quite similar. For the modified fabrics, **QD** deposits were clearly seen onto the surface of fabrics for the all QDs@Q-cotton. Particles in microsize were observed for all modified fabrics, except for QD-4@Q-cotton which recorded larger particles with some agglomerations.

**Fig. 6:**
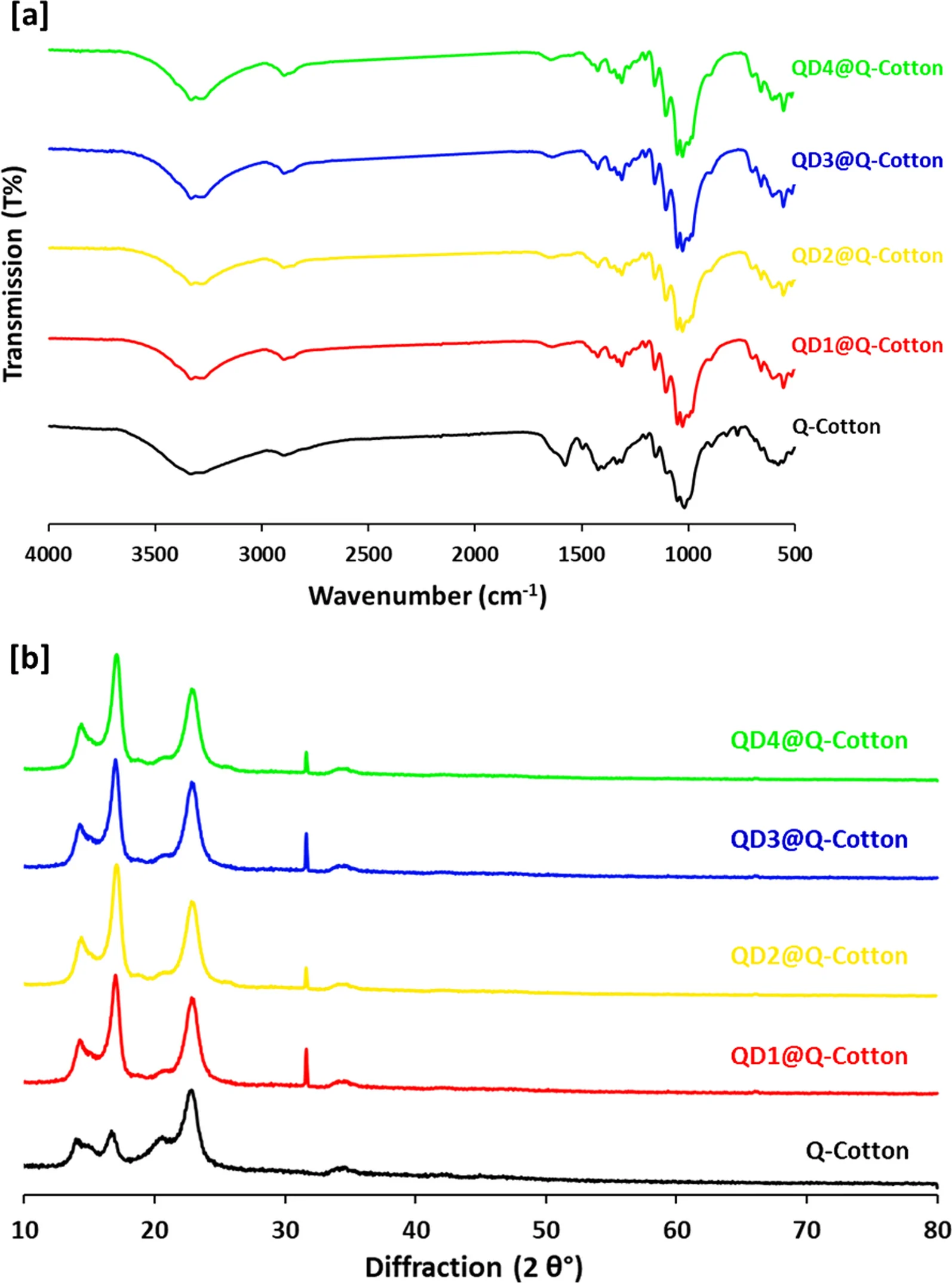
**(a)** FTIR spectra for QDs@Q-cotton fabrics and **(b)** XRD for QDs@Q-cotton fabrics

The chemical interaction between Q-cotton and **QDs** was investigated by infrared spectral results which displayed in Fig. 6a. The Q-cotton exhibited five characteristic transmission bands for the hydroxyl group O–H, aliphatic C–H, N–H group, CH_2_ and the C–C bond, at the wavenumber of 3303, 2892, 1577, 1425 and 1018 cm^−1^, respectively [5, 6]. After immobilization of **QDs**, the intensity of N–H (1577 cm^−1^) group of Q-cotton was significantly decreased due to the interaction with **QDs**. Moreover, two new small bands were appeared at 688 and 557 cm^−1^ which corresponded to the **QDs** molecules. The summarized results supported the interaction of fabrics with **QDs** molecules and further confirmed the formation of QDs@Q-cotton fabrics.

The XR diffractions for the QDs@Q-cotton were measured as shown in Fig. 6b. The Q-cotton exhibited three significant diffraction bands at 2θ° = 14.3θ°, 16.9° and 22.8°, which are assigned for the crystalline structure for cellulose I [2, 54, 90]. After modification with **QDs**, the cotton fabrics showed one additional sharp diffraction peak at 2θ° = 31.6° which could be attributing to the crystal structure of **QD**. The new assigned diffraction is recorded with different intensity depending on the derivative of **QD**. The diffractions for cellulose I were recorded for QDs@Q-cotton which indicating that the cellulose crystallinity was not changed and consequently, the cotton fabrics were not deformed after modification.

### Optical properties of the modified fabrics

**Fig. 7:**
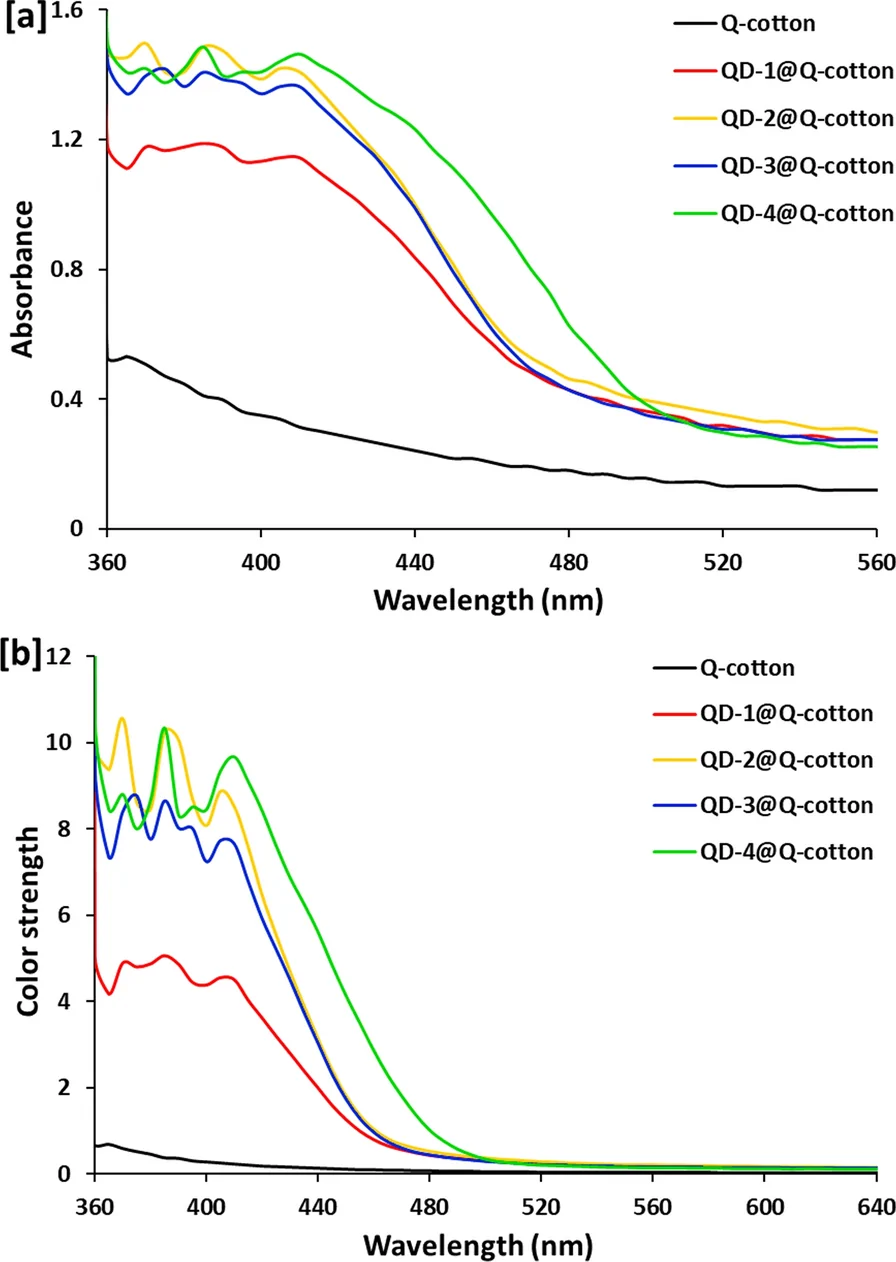
Optical properties; **(a)** absorbance spectra and **(b)** color strength

After modification of cotton with QDs, the color of fabrics was visually changed to yellowish color. Hence, the color data, absorbance and color strength for the QDs@cotton fabrics were measured as presented Table 1 and Fig. 7a & b. The obtained color data declared that the color of fabrics turned from the bale yellow color to dark yellow color after modification with QDs. The absorbance was increased from 0.46 for Q-cotton to 1.17 and 1.45 for QD-1@Q-cotton and QD-4@Q-cotton, respectively (Fig. 7a). Darker yellow color was observed for the QD-1@Q-cotton fabric, while the color strength was raised from 0.8 for Q-cotton to 4.4 for QD-1@cotton and to 10.0 for QD-4@cotton (Fig. 7b). The color data is in agreement with the absorbance data for the solutions of compounds QDs. From the tabulated data in Table 1 it could be declared that; the estimated value of L* for all of the examined samples was followed the order of Q-cotton (80.33) < QD-1@Q-cotton (78.19) < QD-2@Q-cotton (76.14) < QD-3@Q-cotton (78.08) < QD-4@Q-cotton (78.41). Moreover, QD-4@Q-cotton was observed with the highest estimated value of yellowness index to be 85.29. The mechanical properties of the modified cotton were investigated through measurement the tensile strength (Table 1). The tensile strength of fabrics was slightly decreased from 141.8 MPa for Q-cotton to 125.5–131.8 MPa for QD@Q-cotton. After modification with QD, the decrement in tensile strength was ranged in 7.1–11.5%. The reduction in mechanical properties is insignificant and the tensile strength of QD@Q-cotton is quite reasonable for the wearable textile.

**Table 1:** Color measurements and tensile strength for the synthesized QDs@Q-cotton fabrics

Sample	L*	a*	b*	YI E313 [D65/10]	Tensile strength (MPa)
Q-cotton	80.33	0.68	19.84	17.28	141.8
QD-1@Q-cotton	78.19	− 5.29	35.43	59.65	129.1
QD-2@Q-cotton	76.14	− 5.43	39.31	65.79	125.5
QD-3@Q-cotton	78.08	− 7.2	40.46	64.74	130.4
QD-4@Q-cotton	78.41	− 7.24	59.4	85.29	131.7

The yellowness index is measured according to ASTM standard E313 in Daylight

**Fig. 8:**
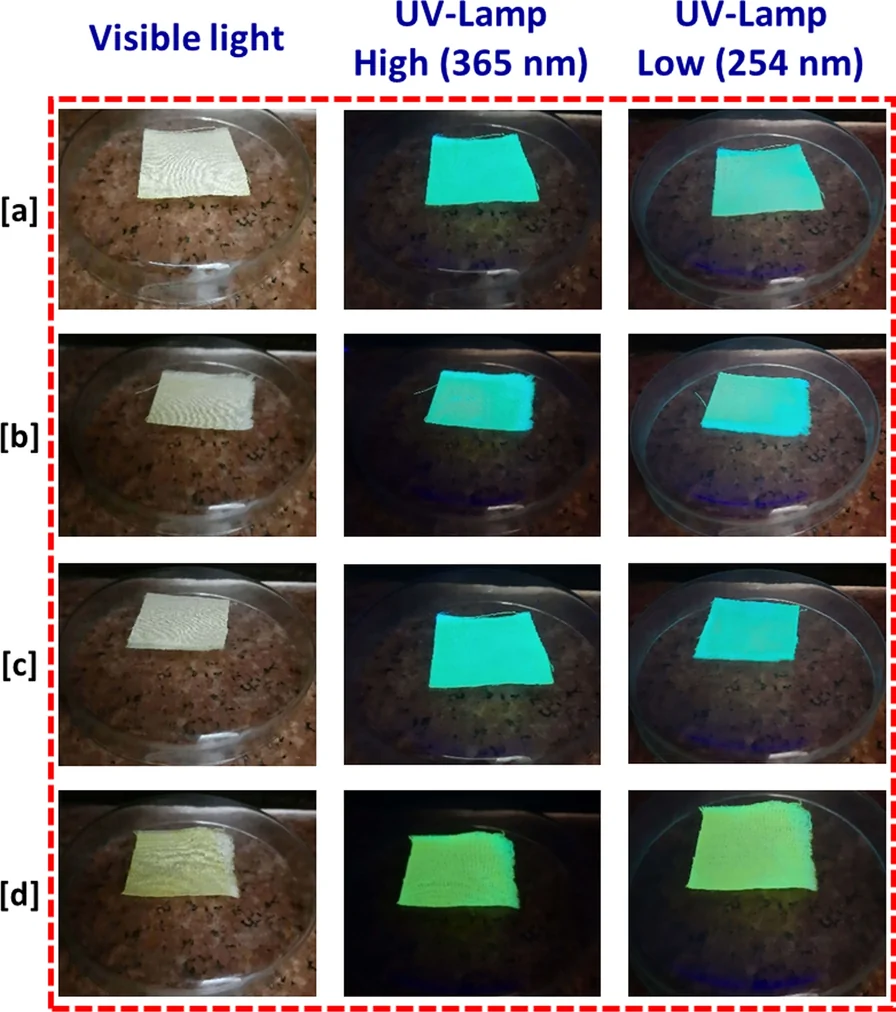
Photographic images for QDs@Q-cotton fabrics; **(a)** QD-1@Q-cotton, **(b)** QD-2@Q-cotton, **(c)** QD-3@Q-cotton and **(d)** QD-4@Q-cotton

**Fig. 9:**
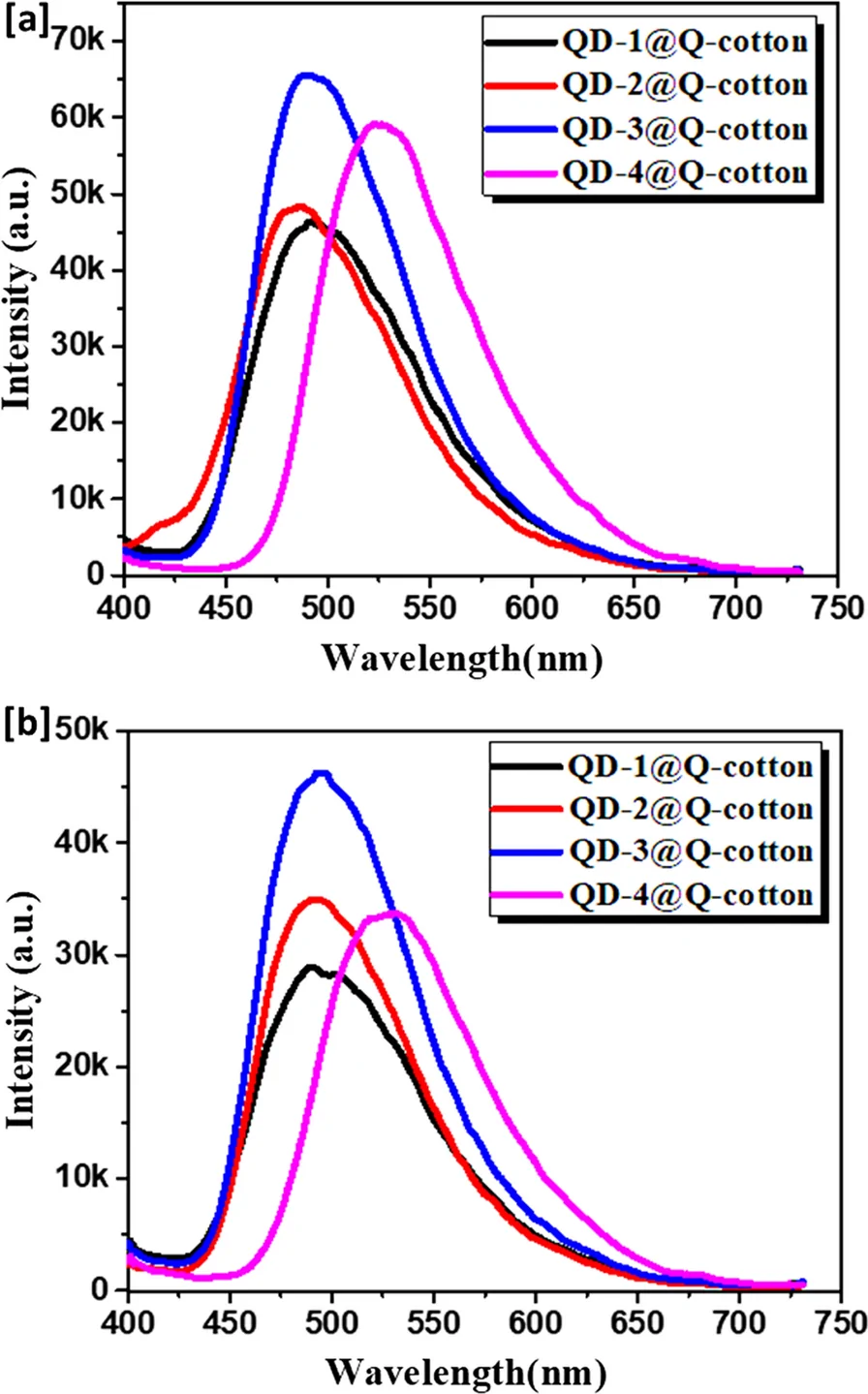
Emission spectra (at 360 nm) for QDs@Q-cotton fabrics; **(a)** before washing and **(b)** after 10 washes

Photos of the modified fabrics in visible and ultraviolet lamp (short wavelength of 254 nm and long wavelength of 365 nm) were taken as displayed in Fig. 8, while the fluorescence emission of fabrics before and after washings were presented in Fig. 9. The all QDs@Q-cotton fabrics released greenish radiation under the UV lamp. The QDs@Q-cotton fabrics showed quite intense fluorescence emission bands at 485–521 nm which are charcaterized for the loaded **QDs** compounds. There was a difference in the place of the fluorescence band which is a fucntion of the type of **QD** with its different substituted group. Compraed with solution, blue shift in the fluorescent emision band was detected for QDs@Q-cotton attributing to the binding interaction between **QD** and cellulose of cotton. The highest intensity of recorded emission band was observed for QD-3@Q-cotton which may be related to the higher affinity of **QD-3** towrads cotton and subsequently helped in high loaded amount of **QD-3** onto fabric.

The fastness property of the functional textiles is quite important factor and required for the wearable materials and consequently the effect of repetitive washings for QDs@Q-cotton on the fluorescent emission was studied. The intensities of the fluorescence emission for the all QDs@Q-cotton fabrics were reduced by small amounts after ten washings (Fig. 9). However, the fluorescence intensity was quite high after washing, reflecting the good durability of the fluorescent QDs@Q-cotton. The decrement in emission intensity is mostly owing to releasing of the applied **QDs** particles from the surface of the Q-cotton.

The fluorescent textiles could be produced by incorporation of different materials such as metal based materials including nanoparticles [41], metal organic framework (MOF) [1], metal doped materials (Zn^+2^ & ZnS) [91–93] as well as fluorescent dyes [94–96]. Due to the effect of soaping, the fluorescent properties (fluorescent spectra and emission intensity) were commonly affected after washing process which considered as a serious problem. As an example, the emission intensities for fabric dyed fluorescent dye rhodamine, fluorescein and acridine orange) were significantly reduced (25–68% reduction) after washing [94]. Subsequently, the obtained fluorescent fabrics by fluorescent dye, aren’t considered a durable in comparison with the QDs@Q-cotton fabrics prepared in the current work. The emission intensities for QDs@Q-cotton fabrics were considerably higher than that for fluorene@polymers/cellulose Zn@carboxymethyl cellulose, AuNPs@fabric and Ln-MOF@fabrics [1, 37, 41, 91–93]. Therefore, the analyzed data revealed that the modification of cotton fabrics by **QDs** is quite interesting to obtain durable fluorescent fabrics with high emission compared to the former studies interesting in fabrication of fluorescent textiles.

### UV radiation blocking

**Fig. 10:**
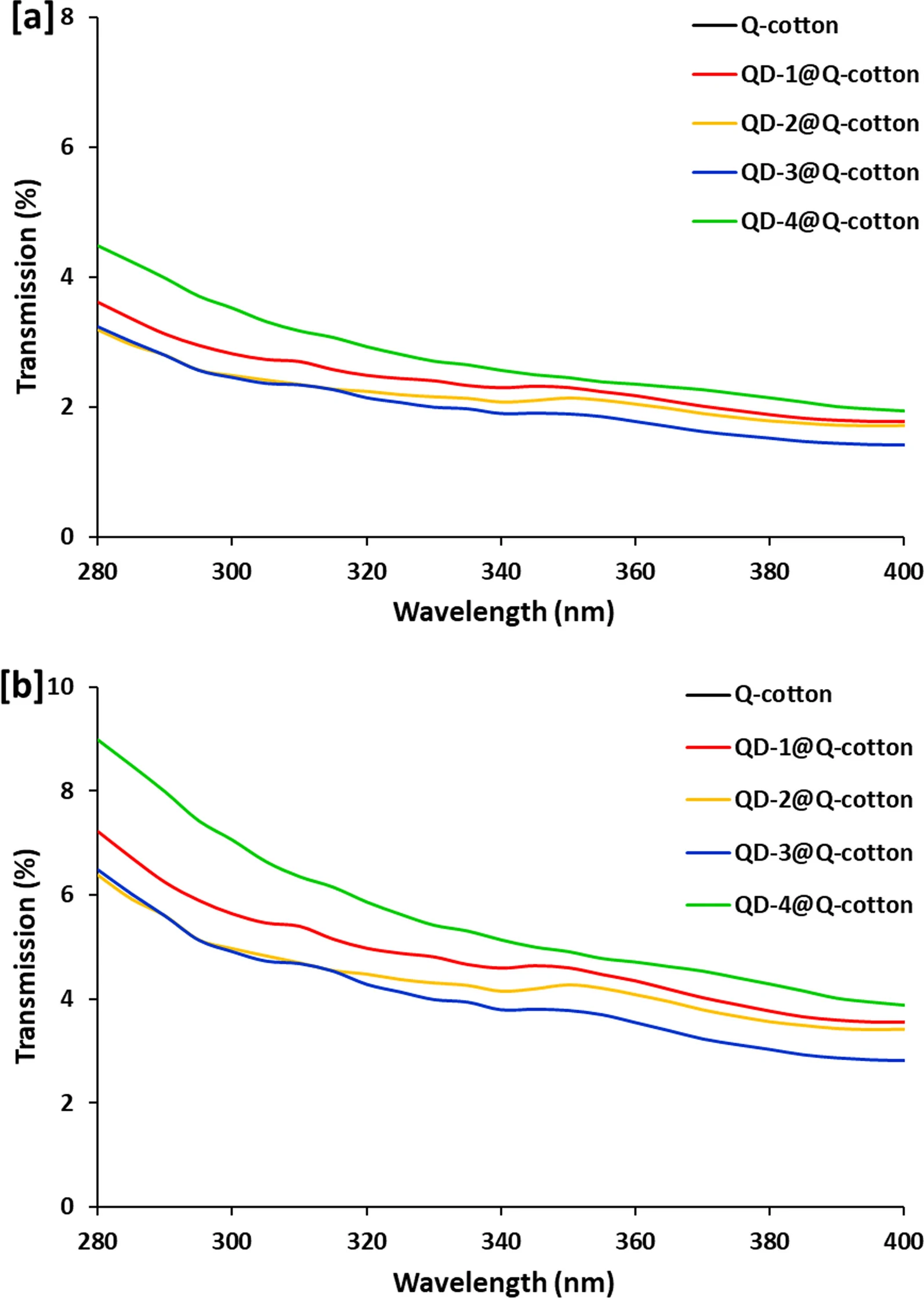
Transmission of UV-radiation over QDs@Q-cotton fabrics; **(a)** before washing and **(b)** after 10 washes

Cellulosic fabrics including cotton are knowing as one from the most likable and applicable textiles owing to their comfortability, breathability and biocompatibility. However, one from the disadvantages of these cellulosic fabrics is that they aren’t UV-protective [51, 97]. Consequently, the modification of cellulosic textile to be UV protective is highly required. Several materials such as metal oxides, metal nanoparticles and MOF were been used for imparting UV-protection character for the cellulosic textiles [5, 7, 43, 51]. Herein, the UV protection character was tested for the obtained QDs@Q-cotton fabrics through measuring the transmission percentage (T%) over the fabrics according to the standard reference procedure of AATCC standard reference method [98]. From the transmission data (Fig. 10), the transmission in the range of 315–400 nm (UVA-T%), the transmission in the range of 280–315 nm (UVB-T%) and the ultraviolet protection factor index (UPF) were all estimated and summarized in Table 2.

**Table 2:** UV-protection properties for the synthesized QDs@Q-cotton fabrics

	Sample	UVA (T%)	UVB (T%)	UPF
	Q-cotton	41.9	45.3	4.4
Before washing	QD-1@Q-cotton	3.9	2.8	28.2
	QD-2@Q-cotton	3.5	2.6	32.0
	QD-3@Q-cotton	3.4	2.3	32.8
	QD-4@Q-cotton	4.8	3.2	23.6
After 10 washes	QD-1@Q-cotton	5.5	3.6	16.5
	QD-2@Q-cotton	4.6	3.1	25.8
	QD-3@Q-cotton	4.9	3.3	21.3
	QD-4@Q-cotton	6.5	4.2	12.6

The cationized cotton didn’t show any protection against UV radiation, as T% was higher than 40 and the UPF index was 4.4 which reflect the insufficient protection of UV. The all QDs@Q-cotton samples showed UV protection with different values depending on the type of **QDs**. The UPF index for QD-1@Q-cotton and QD-4@Q-cotton was 28.2 and 23.6 which are rated as good UV protection. QD-2@Q-cotton and QD-3@Q-cotton exhibited much higher UV-protection (very good protection) with the UPF factor of 32.0 and 32.8. After ten washings, UV protection data was decreased due to the leaching out some of **QDs** to the washing liquor. The washed samples of QD-1@Q-cotton and QD-4@Q-cotton were not UV protective with UPF of 12.6–16.5. While, good UV protection was recorded for the samples of QD-2@Q-cotton (UPF = 25.8) and QD-3@Q-cotton (UPF = 21.3) after 10 washings. The protection from UV radiation for QDs@Q-cotton could be attributed to the reflection of UV radiation by effect of **QDs** micro particles which deposited within the cotton matrix [7, 99].

QDs as heterocyclic compounds were reported to exhibit a high absorption coefficient (ε) in the ultraviolet part of the solar spectral map [100]. According to literature, QDs were hypothesized to act as a protective coating for cotton against photo-motivated damaging by absorbing the harmful solar irradiation [101]. QDs can absorb, above all, irradiation at 290–350 nm. QDs can also transform the absorbed irradiation energies into less harmful thermal energies via the photo-physical processing to involve the ground state and excited state [102]. Light screening, quenching of excited state and scavenging of alkyl peroxyls were reported to be involved for some extent in the QDs mechanism [103]. The UV blocking properties for QDs@Q-cotton is compared with the former studies. The UPF values of QDs@Q-cotton are in the same level for the cellulosic fabrics treated PdNPs [54, 90, 104], SiO_2_/TiO_2_ [105] and MOFs [5, 7]. Meanwhile, the obtained UV blocking properties for QD-2@Q-cotton and QD-3@Q-cotton are much greater than those recorded for cellulose modified with carbon dots [42, 43], metal nanoparticles [90, 104, 106], metal oxides [107, 108] and metal salts [6].

### The microbial activity

The antibacterial activity of QDs@Q-cotton fabrics against gram positive (+) bacteria (*S. aureus*) and gram negative (-) bacteria (*E. coli*). The cationized fabric (Q-cotton) showed quite low bacterial protection with reduction of 19.5% and 16.4% against *S. aureus* and *E. coli*, respectively (Table 3). The antibacterial activity for the fabrics was significantly increased after modification with QDs, while the activity was higher against *S. aureus* compared to *E. coli*. The highest bacterial reduction was observed for QD-3@Q-cotton (89.1% for *S. aureus* and 86.3% for *E. coli*) and the other QDs@Q-cotton samples showed similar antibacterial activities (84.1–87.6% for *S. aureus* and 79.9–86.3% for *E. coli*). The bacterial reduction was decreased after washing attributing to the release of some deposited **QDs** molecules from the surface of fabrics. However, the antibacterial activity for the fabrics was still acceptable (69.3–76.2%) after 10 washings reflecting the reasonable durability for the QDs@Q-cotton against the bacterial pathogens.

**Table 3:** Antibacterial activities for the synthesized QDs@Q-cotton fabrics

	Sample	*S. aureus* (%)	*E. coli* (%)
	Q-cotton	19.5	16.4
Before washing	QD-1@Q-cotton	82.6	80.2
	QD-2@Q-cotton	83.1	80.8
	QD-3@Q-cotton	89.1	86.3
	QD-4@Q-cotton	83.6	79.9
After 10 washes	QD-1@Q-cotton	70.7	69.3
	QD-2@Q-cotton	71.9	70.5
	QD-3@Q-cotton	76.2	74.8
	QD-4@Q-cotton	72.0	69.7

In general, the heterocyclic compounds showed great interest in the medicinal chemistry and the quinazoline derivatives (**QDs**) are considered as one from the most known heterocyclic with the biological properties (such as antifungal, antibacterial, anti-inflammatory, anticancer and anti-analgesic) [109, 110]. Therefore, the antibacterial activity of the QDs@Q-cotton fabrics is attributed to the **QDs** skeleton. The highest antibacterial action for QD-3@Q-cotton is related to the chlorine derivative and this is in agreement with previous work [109]. By comparing with other studies for antimicrobial textiles, the obtained biological activity for QDs@Q-cotton is in the same range as that reported for ZIF-MOF-silicate/fabric [7] and Ce-MOF/viscose [2]. While the estimated data herein is quite greater than that obtained for TiO_2_/fabric, ZnO_2_/fabric [107, 108, 111–113], AgNPs/fabric, AuNPs/fabric and PdNPs/fabric treated natural fabrics [90, 104, 106].

## Conclusion

Multi-functionalized (fluorescent, antimicrobial and UV-protection) cotton as technical textiles were obtained via immobilization of quinazoline derivatives (**QDs**). The preparation of four different **QDs** starting from 2-(chloromethyl) quinazoline-4(3H)-one was chemically investigated by NMR and IR spectroscopy. The obtained **QDs** immobilized within activated cotton (Q-cotton) and microsized **QDs** was seen onto the cotton’s surface. Due to modification with **QDs**, cotton textiles acquired dark yellow color and emitted greenish radiation with significant high emission. QDs@Q-cotton showed good to very good UV blocking and good UV protection after 10 washings. Good antimicrobial activity was recorded for QDs@Q-cotton textile even after washing. The data supported the long-lasting stability of the functionalized textiles against washing. The present study is quite interesting in the textile industry to get durable technical textiles via incorporation of water insoluble hetero compounds, subsequently will open the way for future works in the fucntional textiles by other heteroc compounds. The obtained multi-functionalized textile can be efficiently applied in the soldier clothes and military textiles. In addition, the obtained hetero compounds are furtherly promising in manufacturing of fluorescent materials such as bio-imaging, sensors or smart labeling.

## Supplementary Information

Additional file 1.

## Data Availability

All relevant data are within the manuscript and available from the corresponding author upon request.
