# Supplementary material for: Cutting edge for technical textiles (fluorescent, antibacterial and UV-protective) by incroporation of thienoisoquinoline-quinazoline derivatives
Source: BMC Chem. 2025 Jun 19;19(1):173. doi: 10.1186/s13065-025-01504-3 (PMC12180280; doi:10.1186/s13065-025-01504-3)
Supplement: Supplementary file 1 — Additional file 1. [file 13065_2025_1504_MOESM1_ESM.docx]

**Supplementary file**

**Figure S1:** ^1^H NMR (left) and ^13^C NMR (right) spectra of quinazolines derivatives; **[a]** QD-1, **[b]** QD-2, **[c]** QD-3 and **[d]** QD-4.


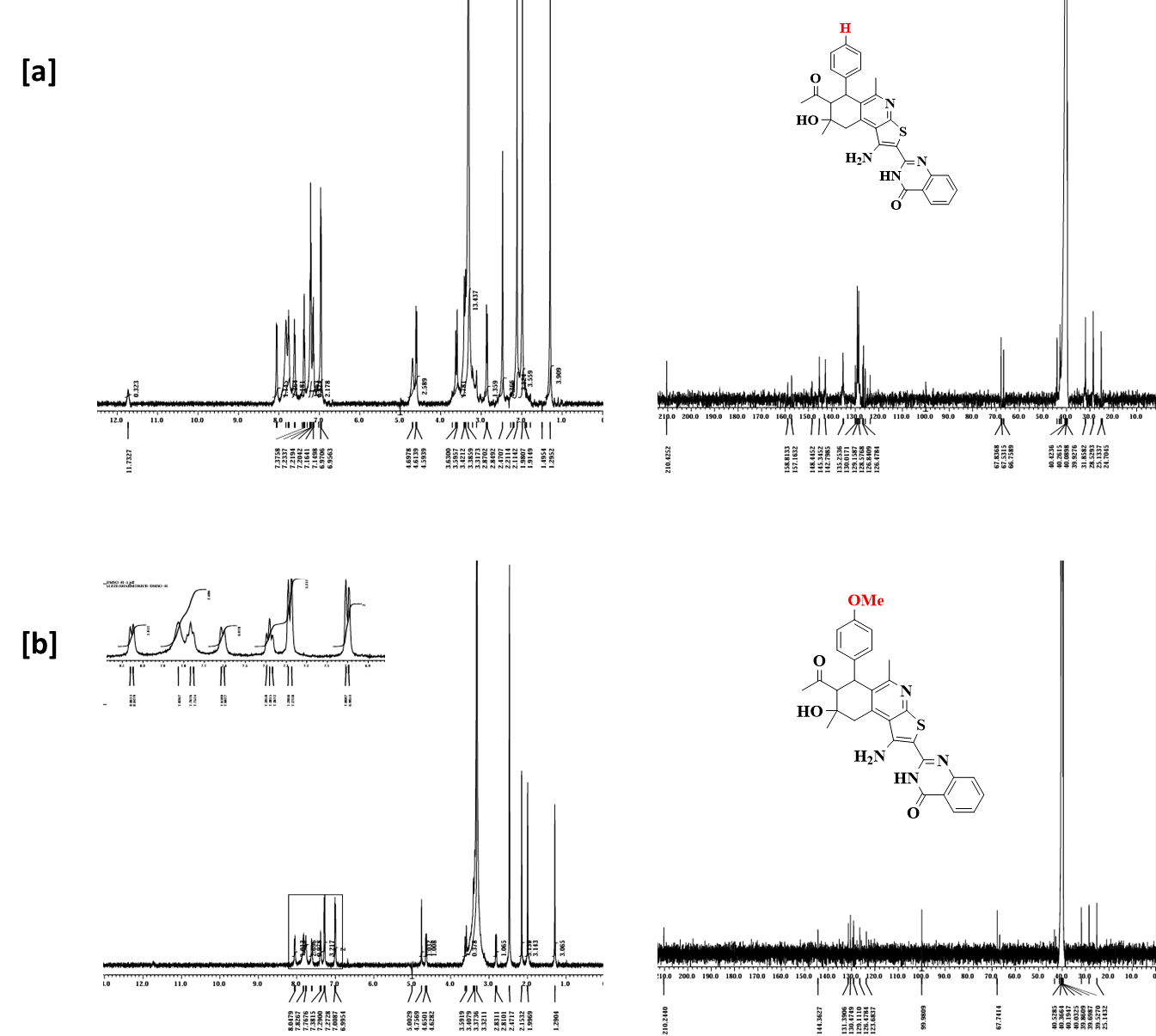


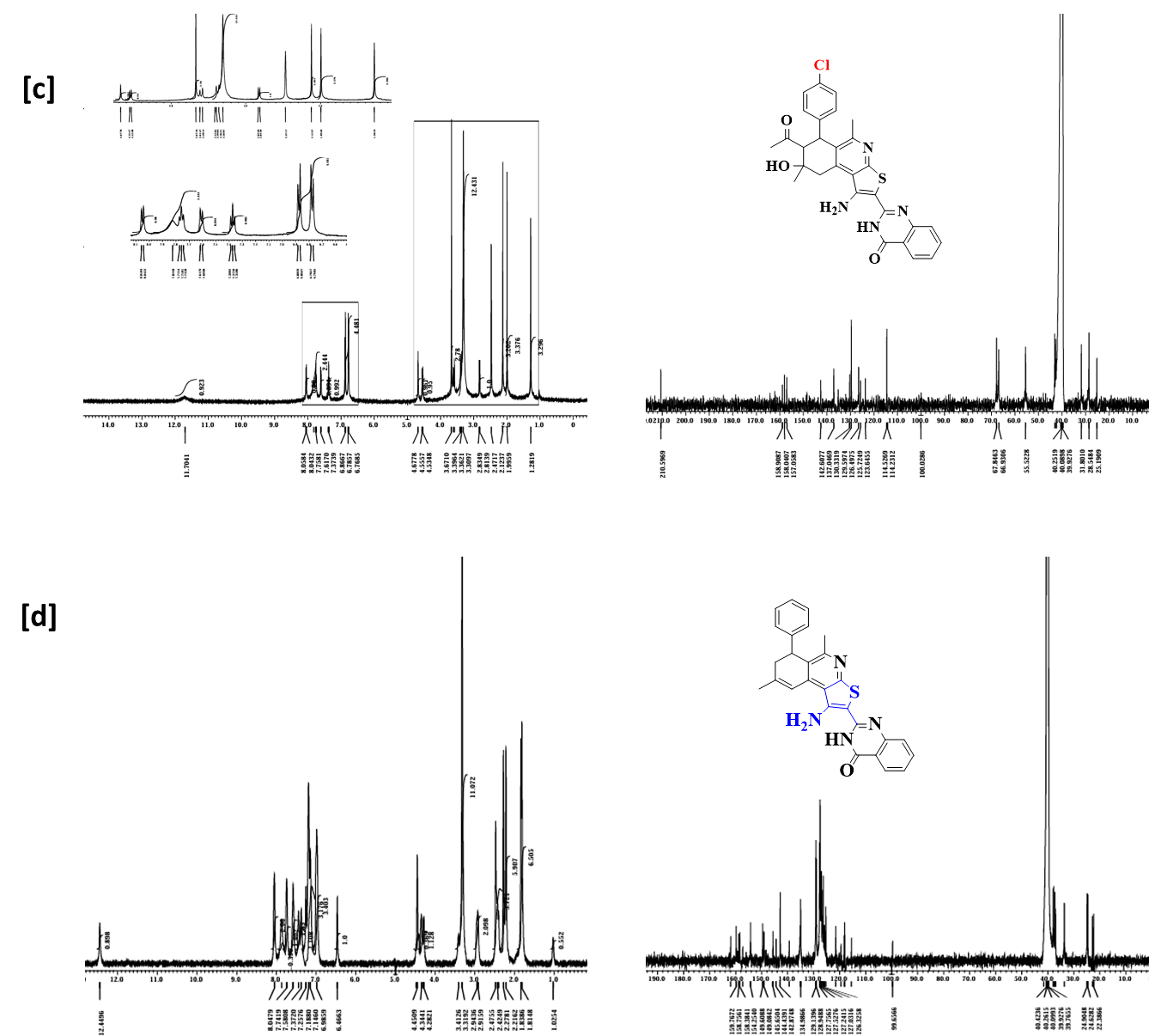


**Figure S2:** Elemental mapping for QDs@Q-cotton fabrics; **[a]** QD-1@Q-cotton, **[b]** QD-2@Q-cotton, **[c]** QD-3@Q-cotton and **[d]** QD-4@Q-cotton.

| **[a]** | **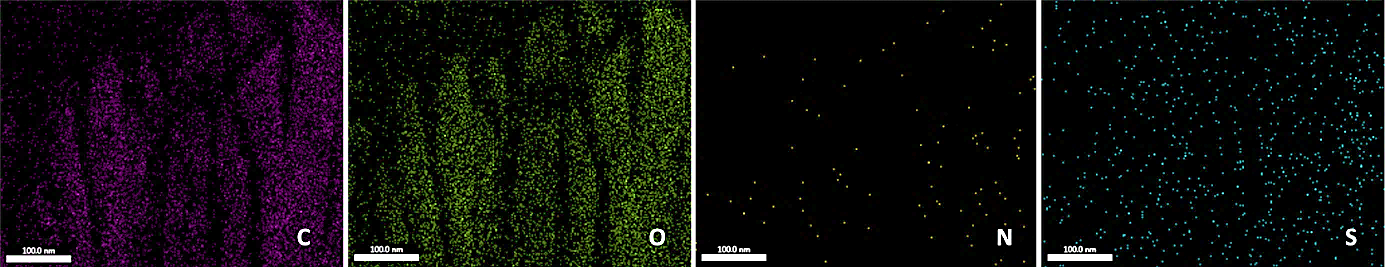** |
| --- | --- |
| **[b]** | **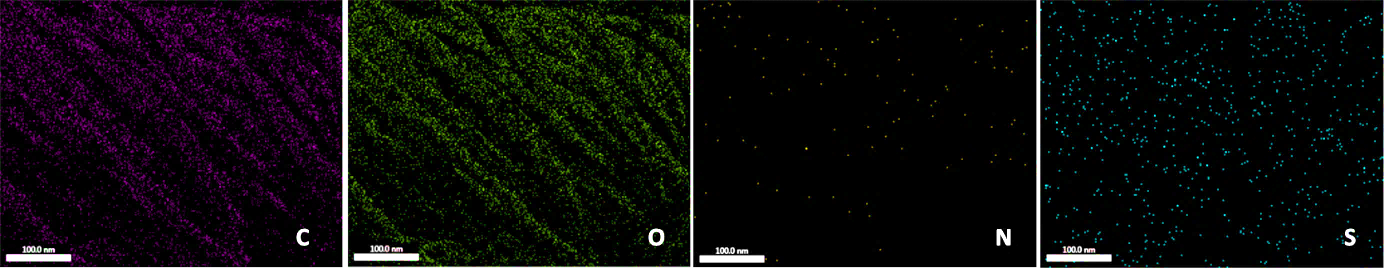** |
| **[c]** | **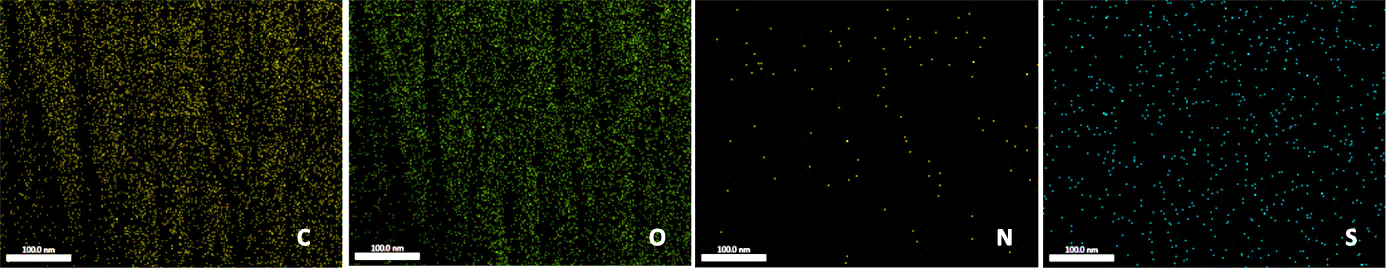** |
| **[d]** | **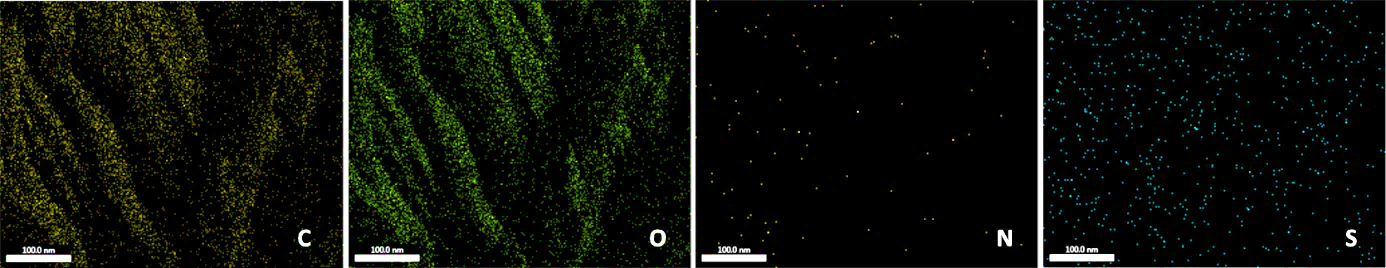** |
